# Occurrence of Alkenylbenzenes in Plants: Flavours and Possibly Toxic Plant Metabolites

**DOI:** 10.3390/plants12112075

**Published:** 2023-05-23

**Authors:** Mario E. Götz, Andreas Eisenreich, Janine Frenzel, Benjamin Sachse, Bernd Schäfer

**Affiliations:** German Federal Institute for Risk Assessment, Department Food Safety, Max-Dohrn-Strasse 8-10, 10589 Berlin, Germany; mario.goetz@bfr.bund.de (M.E.G.); benjamin.sachse@bfr.bund.de (B.S.); bernd.schaefer@bfr.bund.de (B.S.)

**Keywords:** alkenylbenzenes, safrole, estragole, methyleugenol, myristicin, apiol, herbs, essential oils, culinary spices

## Abstract

Alkenylbenzenes are naturally occurring secondary plant metabolites. While some of them are proven genotoxic carcinogens, other derivatives need further evaluation to clarify their toxicological properties. Furthermore, data on the occurrence of various alkenylbenzenes in plants, and especially in food products, are still limited. In this review, we tempt to give an overview of the occurrence of potentially toxic alkenylbenzenes in essential oils and extracts from plants used for flavoring purposes of foods. A focus is layed on widely known genotoxic alkenylbenzenes, such as safrole, methyleugenol, and estragole. However, essential oils and extracts that contain other alkenylbenzenes and are also often used for flavoring purposes are considered. This review may re-raise awareness of the need for quantitative occurrence data for alkenylbenzenes in certain plants but especially in final plant food supplements, processed foods, and flavored beverages as the basis for a more reliable exposure assessment of alkenylbenzenes in the future.

## 1. Introduction

Alkenylbenzenes are known as characteristic flavor constituents of various culinary herbs and spices and are also found in different vegetables and fruits—albeit in lower amounts. They occur as a component of essential oil (EO). Hence, comparatively high concentrations may be found in distillates or alcoholic extracts made of these plants. Such extracts are frequently used for flavoring purposes for various foods and beverages [1].

On the one hand, different secondary plant metabolites, including alkenylbenzenes, are generated in plants—amongst other things—as part of their defense against herbivores and/or pests. On the other hand, animals, as well as humans, possess adapted metabolic capacities for detoxification and are able to eliminate those compounds, e.g., via kidneys. Nevertheless, certain plant metabolites, such as alkenylbenzenes, may pose health risks to humans. Recently, the toxic properties of alkenylbenzenes and the health risks potentially associated with oral exposure to these compounds via the consumption of certain foods were discussed in detail in the context of food safety [2]. In this regard, the genotoxic and carcinogenic potential shown for some well-studied alkenylbenzenes, such as safrole, estragole, methyleugenol, and beta-asarone, is of particular importance. Due to the genotoxic potential of these alkenylbenzenes, the addition of these alkenylbenzenes as such has already been prohibited in the EU via Regulation (EC) No 1334/2008.

In contrast to the abovementioned compounds, the toxicological properties of other alkenylbenzenes, such as myristicin, elemicin, or apiols, are largely unknown [2]. However, structural similarities of those alkenylbenzenes to known genotoxic and carcinogenic alkenylbenzenes, in particular, safrole and methyleugenol, suggest assuming the latter compounds may also have genotoxic properties [3].

Table 1 depicts known chemical structures of alkenylbenzenes that have been found in extracts from certain parts of various plants in highly variable amounts. Most alkenylbenzenes contain a characteristic allylic or propenylic side chain at the benzene ring. The propenylic derivatives may be *cis*- or *trans*-configured. Stereochemical side chain configuration and benzene-ring substituents determine the metabolic fate, in turn influencing the genotoxic activity that is mediated by ultimate reactive intermediates.

For some alkenylbenzenes, e.g., estragole, methyleugenol, and safrole, metabolic activation via 1′-hydroxylation followed by sulfo-conjugation and ultimately, the formation of a reactive carbenium ion has been proven (reviewed in more detail by [2,3]), whilst asarones predominantly form genotoxic side chain epoxide intermediates [4,5,6]. Because of the metabolic specificities of alkenylbenzenes, we recently proposed a potential testing strategy to reliably investigate in greater depth mutagenic properties of different alkenylbenzenes in mammalian organisms in relation to the structural characteristics identified so far [7].

In addition to the abovementioned missing toxicological information, the content of different alkenylbenzenes, such as myristicin, elemicin, or apiols in plants as well as in plant-based foods, is less comprehensively reported [2,3]. In this context, many more alkenylbenzenes with similar chemical structures—and probably comparable toxic properties—may be prevailing in edible parts of plants and, thus, will occur to variable extents in extracts of them, too.

Naturally, plants generate alkenylbenzenes to very varying extents, which depend on species, variety, geographical origin, humidity, solar irradiation, maturation state at harvest, parts of the plants collected, and postharvest processing [2,3]. Moreover, different extracts and oils used for food flavoring purposes can be obtained from various parts of edible plants (e.g., leaves, stems, roots, seeds, and fruits), which also affects the content of alkenylbenzenes in the obtained plant-based materials. Moreover, as alkenylbenzene-containing extracts may be used in very variable amounts, it is important to quantify potentially toxic food ingredients not only in the raw ingredients but especially in the final foods and beverages.

Here, we will summarize and discuss the current knowledge on the occurrence of alkenylbenzenes in EOs and extracts of natural origin from herbs, spices, and some vegetables. In this context, we focused on an underlying literature search on those culinary plants exhibiting alkenylbenzene concentrations of at least 1000 ppm in the corresponding EOs. The obtained results regarding toxicologically relevant alkenylbenzenes in plant extracts and EOs were summarized and presented in alphabetical order as listed in Table 2. Therefore, this review, providing a special focus on culinary spices and those plants that might become of interest for flavoring purposes because of their unique ingredients, is seen complementary to other recent reviews on alkenylbenzenes in the plant kingdom, which offer a wider scope and also address further important aspects [8,9,10].

### 1.1. Occurrence of Alkenylbenzenes in Herbs, Spices, and Some Vegetables

Allspice (*Pimenta dioica* (L.) Merr., Myrtaceae), also known as pimenta, pimento, and Jamaica pepper, is a tree native mostly to the West Indies, Central America, Mexico, and Venezuela. Allspice, its EO, and its oleoresin (less so) are used in beverages, frozen dairy desserts, candy, baked goods, gelatins and puddings, meat products, condiments, relishes, and others [11]. Methyleugenol (63%) was reported to be the main ingredient of the pimenta berry oil when berries from commercial suppliers in Mexico were extracted by steam-distillation for 2 h at 97 °C using a semi-pilot extractor, but it also contained eugenol (8.3%) [12]. In drastic contrast are results reported by Padmakumari et al. in 2011, which used hydro-distillation of pimenta berries from Jamaica [13]. They reported their pimenta oils contained about 75% eugenol and only 4–9% methyleugenol. The chromatographic results are not shown by these authors. In accordance with these results, however, Nabney and Robinson reported that steam-distillation of dried pimenta berries of Jamaican origin yielded eugenol (69%) as the main alkenylbenzene and smaller amounts of methyleugenol (8.8%) and chavicol (0.3%) as a minor component [14]. Pimenta leaf oils contain eugenol, isoeugenol, and methyleugenol [15]. Later analysis differentiated between alkenylbenzene contents in pimenta dioica leaf oil and berry oil and compared those to data obtained from *Pimenta racemosa* leaf oils. Peyron et al. found up to 9.6% methyleugenol and up to 83% eugenol in pimenta berry oils and only trace amounts (0.3%) of chavicol [16]. In *Pimenta dioica* leaves, up to 4.4% methyleugenol and up to 95% eugenol were detected. They also analyzed the leaves of *Pimenta racemosa,* which contained 11–21% chavicol and 38–75% eugenol. Interestingly, in EOs from the plant *Eugenia caryophyllata,* which is usually harvested in Africa and Indonesia, almost no methyleugenol but up to 95% eugenol and eugenyl acetate (up to 7%) were found by Peyron and colleagues [16]. There are further reports on EO components from different regional origins on Carribean Islands and Central America of *Pimenta dioica* trees. The amounts of methyleugenol in EO from pimenta berries is dependent on the individual tree and time of harvest [17]. Thus, EOs from *Pimenta* berries on the market are presumably characterized by variable amounts of methyleugenol.

### 1.2. Anise and Star Anise

Anise (*Pimpinella anisum* L., Umbelliferae, or Apiaceae) is an annual herb native to eastern Mediterranean regions. Anise is endemic (indigenous) to Asia Minor, Egypt, and Greece and is cultivated, e.g., in Turkey, Russia, South Africa, America, and China [18]. However, even with the short season and rather cool climatic conditions in Alberta (Canada), *Pimpinella anisum* can be cultivated [19]. Steam-distillation of the dried ripe fruits (seeds) yields an oil that is rich in phenylpropanoids with *trans*-anethole as major component (79–90%) and, depending on the origin of the anise seeds, as well as on the extraction duration and techniques applied, varying amounts of estragole (1.4–7.1%), *pseudo*-isoeugenyl-2-methylbutyrate (1.2–7.4%) methyleugenol (0.1–0.2%), *cis*-anethole (0.2–0.6%) and isoeugenol (0.03–0.05%). This was quantified using gas chromatography (GC)–mass spectrometry (MS) in conjunction with nuclear magnetic resonance scans to identify unknown preparatively separated fractions [20]. Variations in the EO composition of *Pimpinella anisum* fruits obtained from different geographical regions of Europe were determined by Orav et al. using capillary-GC and GC-MS techniques [18]. The EO content of the samples ranged from 10 mL/kg up to 54 mL/kg. A total of 21 compounds were identified, and significant quantitative differences were observed among the samples. The major component was *trans*-anethole (77–94%); the other phenylpropanoids analyzed in oils were *trans-pseudo*isoeugenyl-2-methylbutyrate (0.4–6.4%) and estragole (syn. methylchavicol) (0.5–2.3%). The highest content of *trans*-anethole (>90%) was found in the samples from Greece, Hungary, Scotland, Lithuania, Italy, and Germany (two samples). EO of anise seeds from Estonia was rich in *trans*-*pseudo*-isoeugenyl-2-methylbutyrate (6.4%) [18], but also cis-pseudoisoeugenyl-2-methylbutyrate can be found in aniseed extracts [21]. In *Pimpinella anisum* seeds and in other *Pimpinella* species, such as *Pimpinella peregrina*, and *Pimpinella saxifraga* that also contain *trans*-pseudoisoeugenyl-2-methylbutyrate, the respective epoxy-pseudoisoeugenyl-2-methylbutyrate was found amongst other esters [22].

Chinese Star Anise (*Illicium verum* Hook. F., Illiciaceae) is a tree widely grown in southeastern Asia (mainly in China and Vietnam). Steam-hydro-distillation or liquid carbon dioxide-mediated extraction of the dried ripe fruits yields star anise oil, which consists mostly of *trans*-anethole (89–92%) [23,24,25] but may also contain *cis*-anethole (0.2–0.7%), estragole (0.5–5.5%) and *trans*-iso-methyleugenol (0.1%) [23,26,27]. The EO from star anise fruits is used in the confectionery trade, to flavor licorice and other candies, in the baking trade to flavor cakes, cookies and biscuits, and in the liqueur industry.

In contrast, the smaller and somewhat deformed dried ripe seeds and the EOs of which from Japanese Star Anise (*Illicium anisatum* L., botanical synonym *Illicium religiosum* Sieb. et Zucc.) are highly poisonous mainly because of its anisatin content [28]. The oil of Japanese Star Anise fruits, *Illicium anisatum* was analyzed by gas chromatography. The major alkenylbenzenes were methyleugenol (9.8%), safrole (6.6%), myristicin (3.5%), and to a minor extent *trans*-anethole (1.2%) [29]. Thus, medicinal and food control authorities must ascertain that Japanese star anise is not present in products containing anise and that EOs destined for the preparation of foods are free of Japanese star anise extracts.

### 1.3. Basil

Sweet Basil (*Ocimum basilicum* L., Labiatae, or Lamiaceae) is nowadays cultivated in many countries around the world, originating probably from Africa and tropical Asia. The chemodiversity of *Ocimum* species is large, and *Ocimum* species have been analyzed for their EO composition in over 50 countries [30,31]. The EO is basically generated from dried leaves and stems (aerial parts of the plant) by steam distillation. In a systematic study of EOs obtained from the aerial parts of seven varieties of *Ocimum basilicum*, it was found that basil oils may contain, relative to other identified components, high amounts of methyleugenol (9.3–87%) and estragole (0–88% especially in the varieties “Lettuce Leaf” and “Dark Green”) depending on the basil variety, season and the environmental conditions, as well as the maturation state at harvest, such as growth height. A comparison of three EOs from *Ocimum basilicum* originating from Morocco, France, and Italy revealed, however, only very low amounts of methyleugenol (<0.8%) and amounts of estragole less than 32% [32]. Usually, the estragole levels in EOs from leaves of sweet basil increase when water is scarce [33].

Another alkenylbenzene found in nearly all sweet basil oils investigated is eugenol (0–34%). In that study, the varieties of *Ocimum basilicum* examined were very rich in methyleugenol (up to 87%) except in “Lettuce Leaf” (lowest contents of methyleugenol 9.2–15%). The methyleugenol content was dependent on solar irradiance, temperature, and relative humidity as determining factors [34].

Other studies on the chemical composition of *Ocimum basilicum* plants focused on the age and the leaf position at the stem [35], as well as differentiated the EO analysis derived from the flowers, leaves, and stems [36]. Eugenol levels were slightly higher in younger leaves and methyleugenol levels predominated in older leaves, but this phenotype appears to be more affected by leaf position at the stem.

The flowers of basil collected in Turkey contained 58% estragole, 0.2% *trans*-anethole, and only 0.03% methyleugenol. The respective leaves contained 53% estragole, 0.6% *trans*-anethole, and 0.2% methyleugenol. Interestingly the basil stems contained less estragole (16%), trans-anethole (0.1%), and methyleugenol (0.06%), but in addition and exclusively found in stems were dill–apiol (50%), apiol (9.5%), elemicin (0.3%), and low amounts of eugenol (0.1%).

It appears that there is still no full clarity on the biosynthetic pathways of alkenylbenzenes in basil species and the environmental factors influencing the expression of biosynthetic enzymes. As discussed by Vani et al. in 2009, chavicol O-methyltransferase identified in crude protein extracts of sweet basil may be responsible for the conversion of chavicol to methylchavicol (estragole) [37]. Eugenol may be transformed into methyleugenol by eugenol O-methyltransferase, both enzymes most likely use S-adenosylmethionine (SAM) as the methyl donor. However, the formation of estragole and methyleugenol is strongly dependent on the season and solar irradiance. Estragole contents may even reach 81% if leaves of *Ocimum basilicum* are extracted with n-hexane instead of hydro-distillation before analysis with GC-MS technology [37]. Using the same techniques, Vani et al. identified high contents of methyleugenol (36–76%) in n-hexane extracts of another basil species *Ocimum tenuiflorum* (also named *Ocimum sanctum*), mainly grown in India. Already in 1984, high levels of methyleugenol (>77%) in EO from two varieties of Fijian *Ocimum Sanctum* (TULSI) varieties were detected [38]. This also highlights that sample preparation and extraction conditions have an impact on the percental distribution of alkenylbenzenes in EOs and plant extracts.

Exposure to estragole and methyleugenol might be low at common use levels of fresh basil, but there are only a few systematic investigations of alkenylbenzene contents in food preparations of various recipes. Moreover, with the consumption of an EO merchandised as a food supplement or the plants being part of dishes in which basil is prepared together with other culinary oils, consumer exposure to alkenylbenzenes may increase considerably. An example is given by Bousova K. et al., who found estragole at a high concentration of 101 mg/kg “Pesto” product [39]. This traditional dish from Genova, Italy, mainly consists of olive oil, hard cheese, pine nuts, garlic, salt, and basil leaves. Very varying levels of estragole in Pesto preparations have been reported (0.05–19 mg/kg versus fresh basil containing 10–16 mg/kg) [40]. Another study reported levels of 3.2–34 mg/kg estragole in “Pesto Genovese” [41]. The same study additionally reported the occurrence of methyleugenol (23–56 mg/kg) and even myristicin (13–16 mg/kg) and, in one sample, apiol (3.4 mg/kg).

### 1.4. Bay

Bay oil from leaves of *Pimenta racemosa* (Mill.) J. W. Moore, Myrtaceae, growing in the West Indies (Dominica), the Caribbean islands, and middle America, and processed two months after harvesting, may contain up to 66% eugenol [42], about 22% of chavicol, 43% methyleugenol, and 32% estragole [43]. However, not each bay variety contains all of the mentioned alkenylbenzenes. A lemon-scented oil variety may not contain alkenylbenzenes at all [43]. An anise-scented oil contained much more mehtyleugenol (43%) than chavicol (2.0%) but high amounts of estragole (32%) and less eugenol (4.3%) [43]. Because of their distinct aromata, bay oils were formerly used as ingredients for the formulation of seasonings and sauces. Botanically, the lemon-scented variety has been known as *Pimenta acris* (Sw.) Kostel var. *citrifolia*. The anise-scented variety does not appear to have been classified at that time. The West Indian bays are botanically quite distinct from the sweet bay, *Laurus nobilis* L., which grows in Europe and other temperate zones [43]. Comparison of the composition and odor properties of the steam volatile oil of the leaves of the California “bay” tree (*Umbellularia californica*) was first made with other well-known “bay” oils from the leaves of Mediterranean bay (*Laurus nobilis*) and West Indian bay (*Pimenta racemosa*) by Buttery and colleagues [44]. The Californian bay oil analyzed by Buttery et al. contained 5.4% methyleugenol but very low amounts of other alkenylbenzenes [44]. However, the acute toxicity of California bay tree leaf oils in mice was slightly higher than that of the West Indian bay tree leaf oil or the Mediterranean bay tree leaf oil [45]. Bay leaf oil from *Pimenta racemosa* may still be used for the production of bay rum and as a flavor eventually at low levels in various foods on the Caribbean islands and the North of South America. The subspecies variants may contain considerably different ranges of concentrations of alkenylbenzenes. The foliar EOs of *Pimenta racemosa* var. *grisea* (Kiaerskov) Fosb. are dominated by geraniol (0–86%), methyleugenol (0.30–93%), and/or trans-methyl isoeugenol (0–86%). The foliar EOs of *Pimenta racemosa* var. *hispaniolensis* (Urban) Landrum are dominated by 1,8-cineole (0.05–38%), estragole (0–23%), methyleugenol (0–64%). The foliar EOs of *Pimenta racemosa* var. *ozua* (Urban and E. Ekman) Landrum are dominated by 1,8-cineole (47–56%), limonene (3.6–30%), and/or α-terpineol (6.7–15%). Commercial bay oil (*P. racemosa* var. *racemosa)* is dominated by chavicol (<0.01–16%), eugenol (44–69%), and methyleugenol (0–12%) [46].

Sweet Bay (*Laurus nobilis* L., Lauraceae) is a tree native to the Mediterranean region. Its leaves are dried and steam-distilled to yield laurel leaf oil. As for the alkenylbenzenes, laurel leaf oil may contain eugenol, methyleugenol, and elemicin, depending on the habitat and maturation state of the leaves. Old leaves in autumn may contain more lipophilic ingredients relative to leaf weight [47,48]. Sweet bay leaves are used as spice and oils, which might be additives to processed foods, including alcoholic and non-alcoholic beverages, frozen dairy desserts, baked goods, condiments, relishes, and others. A comparison of compositions of the EOs of flowers, leaves, and stems of *Laurus nobilis* was reported by Fiorini and colleagues [49]. EOs were obtained by hydro-distillation over 2 h, performed using 300 g of fresh flowers, 500 g of fresh leaves, 100 g of bark stem, or 100 g of wood stem. Eugenol and elemicin were not found in oils from leaves and flowers but in 7.8% and 0.8% in wood stem oils, respectively. However, methyleugenol has been detected in all oils analyzed, with 11.8% in leaf oil, 3.1% in flower oil, 4.7% in bark oil, and 16.0% in wood stem oil [49]. A similar kind of study was performed later with oils obtained from fresh bay leaves (*Laurus nobilis*) that were collected at the North Black Sea region in Turkey [50]. Total concentrations of iso-eugenol, eugenol–methyl–ether, eugenol, and elemicin were below 1% in young leaves, buds, and flowers and below 3% in older leaves. The habitat of a specific plant plays an important role in product quality. GC-MS analysis of the nonpolar fractions (*n*-hexane extracts) of *Laurus nobilis* leaves showed different compositions of constituents from wild and cultivated plants. Unlike cultivated plants, wild *Laurus nobilis* leaf extracts had a considerably higher content of eugenol, elemicin, and methyleugenol. Contents in wild leaves reached 21% for methyleugenol, nearly 5% for elemicin, and 3.1% for eugenol [51]. There have been several studies on EOs obtained from wildly grown *Laurus nobilis* plants in Iran [52] and France, Algeria, and Tunisia [53,54]. All analytical investigations using different extraction methods showed similar levels of elemicin, eugenol, and methyleugenol in leaves of *Laurus nobilis* as those reported by Conforti and colleagues [51].

### 1.5. Carrot

*Daucus carota* L. subsp. *sativus*, Umbelliferae or Apiaceae. Carrot oils may be derived by steam distillation from the seeds and by solvent extraction of the roots of the common carrot cultivars. Carrot seed oils are claimed to be used as a flavor ingredient in many categories of food products [11]. There are rare indications of the presence of very small amounts of methylchavicol, methyleugenol, and apiols in carrot seed oils originating from some carrot subspecies and varieties. However, some EOs from *Daucus carota* subspecies bear high amounts of myristicin or elemicin. Rare EOs have been, e.g., isolated from the myristicin-rich *Daucus sahariensis* from Algeria [55]. Myristicin has been isolated from leaves (34% of the oil) and from the fruits (44% of the oil) of *Daucus sahariensis*. However, as usual, the total oil yield from seeds is very low, with only 0.5% w/w from the leaves and only 0.03% w/w from its fruits in the latter study. Another report of Mediterranean *Daucus carota* L. ssp. *maritimus* by Jabrane et al. reported a level of 30% of myristicin in the respective root EO [56].

Analysis of EOs obtained from *Daucus glaber* Forssk., harvested in Egypt [57], identified mainly elemicin (33% in fruit oil), and methyleugenol (2.5% in fruit oil). Similar results were obtained when the EOs and supercritical CO_2_ extracts of wild *Daucus carota* L. subsp. *carota* from two different sites in Tunisia was investigated. Elemicin contents ranged from 1.4% up to 35% depending on the extraction method, the geographical region, and the genetic differences of the carrot varieties [58].

In 1969, Harborne et al. screened 100 species of Umbelliferae drawn from 50 genera for myristicin content by thin-layer chromatography but could not detect myristicin in seeds from cultivated and wild *Daucus carota* species [59]. The authors concluded that myristicin seemed to be rare as a seed character within the botanical family of the Umbelliferae. However, EOs from seeds of other Umbelliferae, such as dill, parsley, fennel, and parsnip, may also contain considerable amounts of myristicin besides other alkenylbenzenes.

As well, an essential oil obtained from carrot root (*Daucus carota* L. var. *Sativa*, type Imperator), amounting to 40 ppm of the carrot root, has been analyzed using capillary and conventional GC separation with characterization by mass and infrared absorption spectrometry. Parsley is in the same family (Umbelliferae) as carrots, and in that carrot root oil myristicin was present at 0.4% of the whole essential root oil isolated [60]. The presence of myristicin in carrot root distillate from the carrot variety “Imperator” was confirmed and quantitated by Wulf et al. to range between 0.5 ppm and 15 ppm relative to the carrot mass of individual carrots [61]. Myristicin contents are very variable in carrot roots with respect to different *Carota sativa* varieties. The myristicin contents in leaves and roots appear to correlate negatively [62]. Myristicin appears to be one of the compounds involved in the formation of a bitter taste in carrots, and interestingly, concentrations of phenolic volatiles, including myristicin, seem to be boosted by ethylene post-harvest treatments [63].

### 1.6. Chervil

The volatile oils of Garden chervil (*Anthriscus cerefolium* (L.) Hoffm., Umbelliferae or Apiaceae) contain mainly estragole (75–80%) [1]) and 1-allyl-2,4-dimethoxybenzene [64]. Fresh chervil leaves are used as a domestic spice in soups, sauces, salads, vinegar for salad dressings, and omelets, and to season poultry, seafood, and young spring vegetables (such as carrots). Chervil oils from herbs and fruits are used as flavor ingredients for meat products [11].

### 1.7. Cinnamon

*Cinnamomum zeylanicum* Blume and *Cinnamomum cassia* J. Presl, Lauraceae, are the most known representatives of cinnamon species. The genus *Cinnamomum* comprises about 300 species, at least four of which are sources of spice cinnamon [65]. Cinnamon is used as a spice and flavoring agent worldwide. Many different species of plants are commonly referred to as cinnamon. Cinnamon used as a spice in the understanding of the Western world refers to the dried inner bark of *C. zeylanicum* indigenous to Sri Lanka and southern India.

Other *Cinnamomum* species, *C. cassia* (syn. *C. aromaticum*; Chinese cassia), *C. loureiroi* (Saigon cassia), and *C. burmannii* (Indonesian cassia), commonly known as cassia, are also commonly marketed as cinnamon [66]. Ceylon cinnamon is obtained from *C. zeylanicum.* There is also Indian cinnamon (*C. tamala* (Buch.-Ham.) T.Nees and Eberm.).

The chemical composition of cinnamon depends on many factors, such as botanical source, climatic conditions, and methods of harvesting and production. Leaves, twigs, bark, and root bark may serve in steam distillation to yield cinnamon oils. Ceylon cinnamon leaf oil is a source of eugenol (up to 92%) [67]. Eugenol is by far the major constituent of Ceylon cinnamon leaf oil (87%), whilst, in contrast, the Ceylon cinnamon bark oil contained only 8.8% eugenol [68]. Analysis of *Cinnamomum zeylanicum* leaf, stem bark, and root bark oils indicated 72 compounds, of which 32 have not been reported before in Ceylon cinnamon oils [69]. In that study, only 2% cinnamaldehyde, but 70% eugenol, 2.5% eugenyl–acteate, 2.3% safrole, and 0.1% iso-eugenol were detected in commercial cinnamon leaf oils from 2.5-year-old plants. The stem bark oil had only 2.2% eugenol and only traces of safrole but the highest amount of cinnamaldehyde (75%). The root bark oil contained 0.5% eugenol and 0.3% safrole, and only 0.7% cinnamaldehyde.

Cinnamon extracts and oils are extensively used as flavor ingredients in most major categories of food products, and eugenol appears to protect *trans*-cinnamaldehyde probably by an antioxidative mechanism against thermal decomposition, whilst eugenol is stable at 200 °C up to 40 min [70]. Small amounts of eugenol (0.2–0.5 mg/100 g) were present in cinnamon applesauce and orange juice. Larger amounts of eugenol (18–20 mg/100 g) were present in chai tea, cinnamon cookies, and in cinnamon gingerbread [70]. Other alkenylbenzenes were not looked for in that study.

*C. cassia* is distributed in China, India, Vietnam, Indonesia, and other countries. It is commonly used in traditional Chinese medicine and also as a traditional spice. More than 160 compounds have been separated and identified from *C. cassia* [71,72].

### 1.8. Clove

Dried flower buds (cloves), stems, and leaves from *Syzygium aromaticum* (L.) Merr. et Perry, Myrtaceae, a tree growing in tropical zones of Asia, East Africa, and America, is used to produce clove oils. The EO isolated from the buds of *Syzygium aromaticum* is widely used and well-known for its medicinal properties. Traditional uses of clove oil include use in dental care as an antiseptic and analgesic, where the undiluted oil may be rubbed on the gums to treat toothache. It is active against oral bacteria associated with dental caries and periodontal disease [73]. The steam-volatile constituents of air-dried buds of *Syzygium aromaticum* were examined by gas chromatography, infrared spectroscopy, nuclear magnetic resonance, and mass spectrometry. Eugenol (81%), acetyleugenol (7.3%), and nine other minor components, including chavicol, were identified [74]. The major component of clove oil is usually considered to be eugenol, with β-caryophyllene and lesser amounts of other components such as benzyl alcohol, but the proportions vary widely. The highest concentration in a clove oil from Tunisia following hydro-distillation and gas chromatography was eugenol (89%), and the second most abundant compound was eugenyl acetate (5.6%), followed by β-caryophyllene (1.4%). In view of the known activities of the constituents and the variability of the oil, it is important that the real composition is known [75]. In a clove oil from Egypt, 22 compounds, including eugenol (71%) and eugenyl acetate (7.0%), methyleugenol (0.3%), and estragole (0.4%), were identified [76].

### 1.9. Dill

Native to the Mediterranean region and Asia, dill plants (Southern European dill *Anethum graveolens* L. and East Indian dill *Anethum sowa* L., Apiaceae) are now cultivated worldwide [77]. Oils can be obtained by steam distillation from all parts of the fresh plant. Alkenylbenzenes found in dill oil are dill–apiol, eugenol, anethole, and myristicin in various amounts depending on the species and the geographical region where dill is cultivated. The oils from dill seed (fruit) and dill weed can be differentiated chemically and organoleptically, whereby apiols are predominating in the seed oils. The *Anethum sowa* seeds may be used as a constituent of curry powders (Dey K.L. 1896 cited in [78]). East Indian dill oils may contain up to 12% or 15% dill–apiol [79] whilst, although morphologically similar, the *Anethum graveolens* seeds may contain less dill–apiol [80]. Dill–apiol and parsley–apiol, the latter referred to simply as “apiol(e)” in some references, were only found after 3–4 hrs steam-distillation followed by hexane extraction of dill root distillates, but not of dill green distillates, respectively. Dill roots and greens may contain ppm amounts of myristicin [81]. In contrast, hydro-distillation of *Anethum graveolens* seeds from Saudi Arabia revealed 25% dill–apiol, 0.2% myristicin, 0.2% cis-asarone, and interestingly 0.05% ortho-eugenol, also called 2-methoxy-6-(2-propenyl)phenol or 2-allyl-6-methoxyphenol [82]. The chemical constituents of culinary spices are widely dependent not only on species but also on geographic location, seasonal variation, and methods of extraction and analysis. Uses of dill oils are manifold in many food products, such as dishes for culinary purposes in condiments, relishes, and snacks. Chemically, dill–apiol and parsley–apiol have a common sum formula; however, methoxy-substituents differ with respect to their position at the benzene ring. Dill–apiol was chemically identified as 6-allyl-4,5-dimethoxy-1,3-benzodioxole, whereas parsley–apiol was characterized as 5-allyl-4,7-dimethoxy-1,3-benzodioxole.

Dill–apiol is also found in methanolic extracts of dried leaves of *Perilla frutescens* BRITTON var. *acuta*, Labiatae, used in traditional Chinese medicine [83]. In Iran, dried fruits and leaves of *Psammogeton canescens,* Umbelliferae, are added to soups, cheese, and yogurt for flavoring, and its EO from the aerial parts following hydro-distillation contain up to 15% parsley–apiol and 5% dill–apiol, as determined by GC-MS technology [84]. Some carrot seed oils (from Daucus carota) may also contain up to 3.7% parsley–apiol, and some varieties native to Japan up to 40% *trans*-alpha-asarone (multiple citations in [85]).

### 1.10. Elemi

Steam distillation of the resinous exudates of the trees *Canarium commune* L. or *Canarium luzonicum* Miq., Burseraceae, native to the Philippines and the Maluku Islands (also called Moluccas, an archipelago in Eastern Indonesia), yields elemi gum (oleoresin) and oil. Amongst many other components, these oils contain elemicin. Although elemi oil may be used mainly as a fragrance component [86], there are notions that it might be used as well for food flavoring purposes [87] and in alternative medicine [88].

### 1.11. Fennel

The herb fennel (*Foeniculum vulgare* Mill., Umbelliferae, or Apiaceae) is cultivated in many countries of the world. EOs can be obtained by steam distillation of the dried ripe fruits or other parts of the plant, such as leaves, stems, or roots [89]. The common wild fennel is bitter (subsp. *vulgare* var. *vulgare*), and the cultivated one is rather sweet (subsp. *vulgare* var. *dulce*). EO yields from seeds can be 2–6%, the major constituent of which is usually *trans*-anethole (60–90%) [90,91]. Depending on the extraction methods used, estragole contents typically vary between 3.3% and 5.3% in the aerial parts of the plant [92]. Trenkle found in the aerial parts of the sweet fennel (stems, leaves, and seeds) *trans*-anethole (9.7–55%), *cis*-anethole (0.1–0.8%), and estragole (2.0–3.0%) but no myristicin [89]. However, the fennel roots did neither contain anetholes nor estragole but contained instead dill–apiol (46–63%), myristicin (2.5–10%), and parsley–apiol (0.2%). Usually, European fennel seed varieties yield between 1 and 4% estragole and very high amounts of *trans*-anethole [93]. Some previous studies on fennel fruit EOs have also mentioned estragole chemotypes in variable amounts, where estragole alone dominates the oil or is present together with either *trans*-anethole or fenchone. The estragole contents of the EOs from fruits analyzed in the study by Miguel et al. were much higher (up to 88%) than those often reported in the literature for estragole-rich fruit oils [94]. In 2016, Lawrence reported estragole contents in EOs from fennel fruits of not more than 6.8% [95]. However, in oils produced from the leaves and stems of a variety of *F. vulgare* grown in Ethiopia, Mikre et al. identified estragole contents of up to 88%, indicating the huge differences in EO compositions in fennel EOs on the global market [96]. In a more recent study, Hao and colleagues systematically analyzed nutritional compositions and EO profiles of fruits, umbels, stalks, and roots of one dill and two fennel cultivars [97]. They unequivocally confirmed that there are seeds of cultivars that are very rich in *trans*-anethole and other seeds of cultivars that are very rich in estragole. The roots of fennel and also those of dill can be distinguished from the aerial parts by the myristicin content [97].

The oil from sweet fennel fruits (commonly known as seeds) is used as a flavoring component in many products. Very common, e.g., in Europe, is the consumption of fennel herbal teas. Determination of estragole in infusions from different widely used commercial herbal teas based on *Foeniculum vulgare* seeds by an optimized headspace solid-phase microextraction followed by GC-MS analysis revealed levels of estragole to range within 50–250 µg/L [98], or even reach levels from 241 µg/L to 2058 µg/L in teas from teabags [99]. In preparations of tea extracts from herbal tea mixtures (n = 16) of the fennel–anise–caraway type, estragole contents ranged from 4.0 µg/L to 77 µg/L, whilst *trans*-anethole concentrations ranged from 83 µg/L to 7266 µg/L [100]. Interestingly, one-hour following ingestion of fennel–anise–caraway tea by breastfeeding women, approximately 1% (i.e., 0.13 µg/L milk) of the consumed estragole and up to 5% (i.e., 4.23 µg/L milk) of the consumed *trans*-anethole were found in human milk of lactating mothers [100]. An earlier study observed a mean concentration of 9.9 µg/L *trans*-anethole in the milk of breastfeeding women at two hours following ingestion of a 100 mg *trans*-anethole-containing capsule. Peak concentrations of *trans*-anethole in human milk were 23.2 µg/L [101].

### 1.12. Lemon Balm

Lemon balm (*Melissa officinalis* L., Labiatae, or Lamiaceae) is an herb growing in western Asia and the Mediterranean region. EOs from dried leaves and flowers can be obtained by steam distillation. Eugenol may be a constituent of lemon balm EOs (3.5–4.5%), but it does not appear to always occur in the steam volatile fractions of *Melissa officinalis* leaves [102]. However, eugenylglucoside has been isolated in a pure state from *Melissa officinalis* leaves [103]. In one EO from a plant grown in Germany but not in two Finnish lemon balm samples, estragole was found (6.3%; [104,105]). Balm extract and oil are used in major categories of food products, including alcoholic and non-alcoholic beverages, frozen dairy desserts, candy, baked goods, gelatins, and puddings [11]. To our knowledge, the occurrence of other alkenylbenzenes has not been reported so far in the EO of lemon balm.

### 1.13. Lemon Grass

There are many species in the *Cymbopogon* genus which are cultivated for their oil, but *Cymbopogon winterianus* (Java Citronella) is one such species that is native to Sri Lanka and has been domesticated in India for commercial purposes for years. The wide distribution of the genus *Cymbopogon* is due to its adaptability to diverse climatic conditions. As for oils from lemon grass (*Cymbopogon citratus*) citral is the major component, with myrcene being often the second major component. The lemongrass EO extracted from the leaves is commonly used in different pharmaceuticals, flavors, cosmetics, food preservation, and agriculture industries. Fractional vacuum distillation may be an effective method to upgrade lemongrass EOs and may result in fractions with higher amounts of citral or myrcene [106]. *Cymbopogon flexuosus,* Gramineae, or Poaceae, also known as East Indian lemongrass, may contain considerable amounts of citral and geraniol. However, oils derived from certain varieties of which may contain up to 20% methyleugenol [107]. Although not very common in lemon grass oils, Guenther reported that Java-type lemon grass might contain eugenol, methyleugenol, and chavicol [108]. In 1973, Wijesekera investigated various species and found only traces of methyleugenol in Java-type lemon grass (*Cymbopogon winterianus* Jovitt.) [109]. However, in oils from the Cylon-type grass (*Cymbopogon nardus*) 7.2% iso-methyleugenol and 1.7% methyleugenol were detected [110]. Thus, it may be of relevance to determine the actual alkenylbenzene contents in lemon grass oils, especially if intended to be used for food flavoring purposes [111].

### 1.14. Lovage

*Levisticum officinale* W.J.D. Koch (lovage), Apiaceae, is a plant native to south-Western Asia and southern Europe. Among the several species of aromatic plants used in culinary products, *Levisticum officinale* was once recognized, being considerably used by the condiment industry and by households in soups, stews, and meat dishes [112].

A recent comparison and classification of parsley, lovage, basil, and thyme EOs based on their chemical composition showed that β-phellandrene was the major component identified in parsley and lovage EOs, estragole was the major component in basil EO, and p-cymene was the major component in thyme EO. Except for 0.3% anethole, there were no other alkenylbenzenes identified in lovage EO by the study authors [113]. Recently, a comprehensive study on the nutritional, chemical, and bioactive properties of the edible aerial parts (leaves and stems) of a Portuguese-grown sample of *Levisticum officinale* was performed. The chemical composition of the EO extracted from the edible aerial parts (leaves and stems) of the Portuguese *Levisticum officinale* by hydro-distillation did not reveal alkenylbenzenes in this culinary herb [114].

### 1.15. Nutmeg

Seeds of a tropical tree indigenous to the Maluku Islands of Indonesia (*Myristica fragrans* Houtt., Myristicaceae) consist of kernel and aril. The Maluku Islands, or the Moluccas, are an archipelago in eastern Indonesia. Mace is the red lacy covering (aril) surrounding the seed and separates the seed from the outer husk (the pericarp). The dried kernels of the ripe seeds are called nutmeg. When the nutmeg powders are hydro-distilled, about 2.4% of crude oil can be obtained, according to Du et al. [115]. These oils are rich in alkenylbenzenes, such as eugenol (20%), methyleugenol (17%), iso-methyleugenol (17%), myristicin (2.3%), as well as safrole (1.6%), and elemicin (1.7%). In contrast, oils from dried kernels of *Myristica fragrans* originating from Sri Lanka may contain almost no eugenol (0.2%), or methyleugenol (0.6%), nearly equal amounts of safrole (1.4%) and elemicin (2.1%), but higher levels of myristicin (4.9%, [116]). Numerous reports on the composition of nutmeg oils have been published over many years. On the one hand, it is the storage of ground powders (e.g., by [117]; and others: see compilation by [118]), but on the other hand, it is also the geographical origin that determines the volatile compositions of nutmeg extracts as Baldry et al. recognized in 1976 [119]. They showed high variabilities in alkenylbenzene contents, with myristicin ranging from 0.5% to 12%, safrole from 0.1% to 3.2%, elemicin from 0.3% to 4.6%, methyleugenol from 0.1% to 1.2%, and eugenol from 0.1% to 0.7% in different nutmeg oils from West India and South East Asia. The main components of nutmeg EOs from Grenada, Sri Lanka, and East India were compared in 2006 by Reeve [120]. In those EOs, safrole contents ranged from 0.17% up to 1.7%, myristicin from 0.52% up to 10%, and elemicin from 0.37% up to 2.1%. Recent analysis of nutmeg leaves, mace and kernel oils revealed that the highest amounts of myristicin can be found in oils from leaves and mace, of safrole and methyleugenol in mace oils and astonishingly also of γ-asarone in leaf and mace oils from nutmeg trees grown in the Western Ghats of India [121].

Mace powders and mace oils contain similar constituents as kernel powders and kernel oils except for eventually 2–3 times higher levels of myristicin, as demonstrated by preparative thin layer chromatography followed by liquid- or gas-chromatographical methods [122].

Nutmeg and mace are used as domestic spices and as flavor ingredients in many food products such as in gelatins, puddings, sweet sauces, baked goods, meat, fish, pickles (processed vegetables), candy, ice cream, non-alcoholic and alcoholic beverages, such as eggnog, and may exist in even more food commodities of various food categories (this issue was reviewed in detail elsewhere [123,124]). In addition, plant food supplements are globally available containing nutmeg seed powders or nutmeg oils to very varying extents [125]. Analytical methods for ground nutmeg, wine, and beer spices, and many food commodities are in place utilizing ultrasonic-assisted extractions as well as solid phase extractions followed by GC-MS analysis [126]. Another study reported 17 mg myristicin per gram of dried nutmeg powder following 12 h methanol extraction at 50 °C [127]. In 22 powdered Indonesian nutmegs extracted with boiling methanol, myristicin accounted for 1.4–2.9%, and safrole accounted for 0.12–0.43% of nutmeg powder [122]

It is important to mention that in addition to the well-known alkenylbenzenes that are usually identified in nutmeg oils, many other compounds, such as diarylpropanoids, were found in *Myristica fragrans* kernel- and aril-extracts [128,129,130,131]. With advanced analytical technologies, more than 100 substances have been identified (recently reviewed by others [132,133,134]). Some of which do also carry alkenyl groups and may or may not contribute to toxicity. Nowadays, nutmeg consumption of 1.5 g and less of nutmeg powder by human adults can be monitored via the detection of urinary metabolites of safrole, myristicin, and elemicin [135,136].

### 1.16. Parsley

Garden parsley (*Petroselinum sativum* Hoffm. and *Petroselinum Crispum* Mill., Umbelliferae or Apiaceae) is a well-known and widely used domestic herbal spice in Europe and Northern America, either freshly used as culinary garnish or as dried leaves nearly to any kind of food (up to 1.9%) [137]. Parsley seed oil, herb oil, and root oil have different flavors and vary in their composition also, depending on the respective parsley varieties. The composition of the fruits, leaves, and roots of some parsley varieties was thoroughly analyzed by Franz and Glasl already in 1976 [138]. Parsley seed oils may contain parsley–apiol (0.6–79%), myristicin (20–94%), and tetramethoxyallylbenzene (0.1–22%). Parsley leaf oils may contain 3.1–92% myristicin and parsley–apiol (0.7–54%) but no tetramethoxyallylbenzene. Parsley root oils may contain no tetramethoxyallylbenzene but myristicin (6.6–30%) and parsley–apiol (50–77%). In parsley root oils, parsley–apiol always prevails in higher concentrations than myristicin, whilst parsley leaf oils have been found to be in most cases richer in myristicin [139]. Hydro-distillation of parsley seeds for 1 h via a modified Clevenger trap method yielded the highest amounts of alkenylbenzenes in the resulting oils, with about 60% myristicin in a parsley seed oil from Yugoslavia and 22% parsley–apiol in a parsley seed oil from Turkey. The overall yield of EOs from parsley seeds in this study was 0.04% and 0.2%., respectively [140]. When calculated in relation to fresh parsley, parsley–apiol, and myristicin concentrations were reported to amount to 1275 ppm and 1435 ppm, respectively [141]. In 1998, Masanetz and Grosch reported highly variable amounts of myristicin in fresh parsley leaves, ranging between 3.6 ppm and 526 ppm [142]. GC-MS/GC-FID analysis of *Petroselinum crispum* EOs from Mauritius identified the most dominant components to be myristicin (40%) and elemicin (1.7%) [143].

Parsley and dill teas can be purchased without restriction, and recently, levels of alkenylbenzenes in such herbal teas were investigated. Myristicin, apiols, and also methyleugenol have been detected to very varying extents in dry tea samples or in hot water herbal extracts containing parsley or dill leaves or seeds or being a mixture with other herbs [144]. It has been estimated that health concerns exist if food supplements containing parsley and herbal teas containing large amounts of dill are consumed regularly [145].

### 1.17. Pepper

One of the widely distributed plant genera in pantropical regions is the genus *Piper*. *Piper* plants are also known under the common name “pepper”. The presence of oil cells in the structures of almost all *Piper* species places them in the group of aromatic plants [146]. Pepper fruits (*Piper nigrum* L. and others, Piperaceae), a perennial woody vine type plant originating from India and Southeast Asian countries, are extensively used as a domestic spice and as flavoring components in many food products. Different types of black peppers are available, having different colors. The most commonly known peppers are black and white peppers. Black pepper is normally produced by cooking the unripe fruits of the pepper plant in hot water. White pepper is produced from the same plant. After soaking for a few days in water, the berry skin is then rubbed off, resulting in the naked seed. The seeds are then dried. Black pepper is naturally distributed in India, where the Western Ghat forests are rich in this plant. The biodiversity hotspots in this area are reported to be the only known existing source of wild *Piper nigrum* germplasm in the world [146]. The chemical compositions of *Piper nigrum* fruit volatile oils from the Aromatic Plant Research Center (APRC) collection were investigated recently by Dosoky et al. [147]. They found that in EOs from fruits of *Piper nigrum* the *trans*-β-caryophyllene content dominates, while no alkenylbenzenes were reported. On the other hand, modern multi-analyte methods revealed considerable amounts of alkenylbenzenes in fruits from various *Piper* species, including *Piper nigrum* [148]. This study appears as being the first in Europe to determine alkenylbenzenes in pepper fruits as sold on the European market. They found contents of eugenol (10–120 mg/kg), *trans*-anethole (11–43 mg/kg), and estragole (2.2–46 mg/kg) to be the most abundant alkenylbenzenes. Minor amounts were detected for *trans*-iso-eugenol (0.69–3.6 mg/kg) and safrole (0.2–3.0 mg/kg).

EOs from *Piper* species contain variable amounts of alkenylbenzenes. The respective pattern of alkenylbenzenes and their occurrence level depends on geographical region and environmental factors (high humidity and temperatures between 10 °C and 40 °C are needed for growth). Amongst many other compounds, the polar fraction of steam-distilled Ceylon black pepper berry oil contained some eugenol, methyleugenol, safrole, and myristicin [149,150].

Investigations of volatile aroma compounds of the EOs of dried black pepper fruits (*Piper nigrum*) and black and white “Ashanti pepper” (*Piper guineense*) from Cameroon by means of solid-phase microextraction (SPME) headspace GC-MS-technology revealed the presence of safrole, methyleugenol, eugenol as well as of sarisan (which is structurally very similar compared to myristicin, previously described as presumably being genotoxic [151]). Occurrence levels in all of the berry oils were below 3%. Elemicin and myristicin were only detected in oils from berries from *Piper guineense* in concentrations below 1% [152].

The composition of the total volatile oil from the fruits (berries) of “Ashanti pepper” (*Piper guineense*) originating from Nigeria was investigated in detail by means of GC-MS [153]. Their steam-distilled oily extracts of ripe fruits (berries) contained very high amounts of myristicin (17%), sarisan (16%), safrole (4.8%), elemicin (3.9%), and methyleugenol (1.5%), as well as low amounts of iso-elemicin (0.2%), dill–apiol 0.09%), and eugenol (0.07%). Remarkable is the presence of high amounts of sarisan and myristicin that have not been reported in berries from other *Piper* species so far.

Some recent comprehensive reviews summarized the findings on volatile constituents of EOs from the leaves of various *Piper* species, with some of them containing high amounts of myristicin, safrole, and methyleugenol and others containing nearly none of them [146,154,155].

EOs of *Piper* leaves in which considerable amounts of phenylpropanoid compounds were identified are *Piper caninum, Piper auritum, Piper hispidinervum*: (safrole); *Piper aduncum*: (dill–apiol), *Piper divaricatum*: (safrole) [156], *Piper betle*: chavibetol; *Piper patulum*: 1,3,5-trimethoxy-2-propenylbenzene; *Piper klotzsdhianum*: 2,4,5-trimethoxy-1-propenylbenzene; *Piper marginatum*: *cis*-asarone [146]. Sarisan was identified in leaf oils from *Piper callosum* Ruiz and Pav. from Peru and *Mechelia montana* Blume from India [157]. In extracts of dried leaves of *Piper sarmentosum* from the Philippines γ- and α-asarone, sarisan, as well as the very rare exalatacin were identified [158]. Sarisan was earlier described as an isomer of myristicin by Kumamoto and Scora [159] when isolated from leaf oil of *Beilschmiedia miersii* but also found up to 39% in EO from *Piper solmsianum* from Brazil [160], and in addition to safrole (18%), up to 74% in *Piper affinis hispidinervum* [161], and up to 13% in *Piper aduncum* from Bolivia [162]. Moreover, parsley–apiol was reported to be a constituent of EOs from Indian *Piper brachystachium* and *Piper angustifolium* [163]. Amongst safrole and γ-asarone, exalatacin, sarisan, carpacin, croweacin, as well as iso-croweacin were occasionally identified to various amounts in leaf oils from *Crowea exalata* and *Crowea saligna* that are native only to the Australian continent but instead of belonging to *Piperaceae* they are classified as *Rutaceae* [164].

The EOs of the leaves of three species of *Peperomia* from the Amazon, Brazil—this genus belongs to *Piperaceae*, too—were hydro-distilled, and 96 volatile constituents were identified by GC and GC-MS [165]. Therein, very variable amounts of myristicin (0.3–7.6%), elemicin (0.2–1.6%), dill–apiol (0.1–55%), and parsley–apiol (0.95–3.2%) were reported. The aromatic compounds elemicin, myristicin, parsley–apiol, dill–apiol, and in some cases also safrole, have also been found in the Amazon *Piper* species. Safrole content in *Piper rotundifolia,* for example, may amount from 0 to 24% [166]. The EO of *Piper subespatulata* contains up to 49% safrole [167]. For instance, *Piper callosum*, *Piper hispidinervum,* and *Piper marginatum* contain high amounts of safrole (64–98%) [168], and *Piper aduncum* has very high amounts of dill–apiol (32–97%) [169]. The *Peperomia inaequalifolia* EO has safrole (32%), 11-H-himachal-4-en-1-ol (25%), and myristicin (13%) as its main chemical constituents [170]. *Peperomia pellucida* EO presents as major compounds carotol (27–32%), dill–apiol (25–30%), and pygmaein (5.5–11%) [171]. The relative chemical composition of EO derived from fresh or dry material from the whole plant devoid of spikes of *Peperomia circinnata* Link var. *circinnata* was different depending on the harvest period. In total, 38 compounds in the EOs extracted in different seasonal periods were identified, with elemicin concentrations ranging between 1.1 and 22%. The highest concentrations of elemicin were present in EOs extracted in July and January, with 14–22%, marginal levels in September (up to 1.1%), and even full absence in November, March, and May. In addition, a subgroup obtained from fresh spikes (January, March, and May) was characterized by the presence of methyleugenol (27–32%) instead [172].

This short overview speaks in favor of a need for a much broader analysis of alkenylbenzenes in pepper fruits and leaves than performed so far, but depending on the season of harvest. In addition to anethole, estragole, eugenol, safrole, and methyleugenol, other alkenylbenzenes, such as myrisicin, elemicin, parsley–apiol, dill–apiol, sarisan, exalatacin, asarones and their iso-forms may have to be taken up into the analytical portfolio for pepper product surveillance if alkenylbenzene exposures of consumers through pepper species are to be properly addressed. Having a high probability of exerting adverse health effects, myristicin and estragole have at least been proposed for future food monitoring activities in spices and herbs [173].

### 1.18. Purple Haze

*Agastache foeniculum* (Pursh) Ktze. (=*Lophanthus anisatus* Benth.), Labiatae, is a perennial, EO-containing plant native to North America. Natives have used this plant for preparing beverages, as a spice in foods, and against colds. The composition of the EO of *Agastache foeniculum* is hardly known. The aerial parts of this plant contain 0.1–0.3% EO. Up to date, estragole (40–93%) and methyleugenol (28–44%) have been reported as the main components of the oil. However, if *Agastache foeniculum* of Canadian origin was cultivated in Finland, methyleugenol contents were marginally low (0.2%) whilst estragole contents were in the expected range at about 75% [104].

### 1.19. Tarragon

Tarragon (*Artemisia dracunculus* L., Compositae, or Asteraceae) also exists in various varieties. The well-known French tarragon is steril, whereas the Russian variety produces seeds and is a perennial herb. The leaves are used to produce EOs via steam distillation. Estragole is often reported to be the main component of the Western European tarragon oils (69%) [174] and 60% [175], with very low amounts of methyleugenol (0.5%) [175]. Usually, estragole levels in tarragon oils vary between 60% and 75% [176] but can also be lower (17%) [177], in this case, with higher amounts of methyleugenol (29%). Alkenylbenzene components in tarragon oils may vary much with the season, geographic location, and with the process of steam distillation. Other components of the group of alkenylbenzenes could be found mainly in the Russian tarragon variety produced from seeds, such as methyleugenol (9.6–28%), *trans*-iso-methyleugenol (0.08–1.4%), and astonishingly high amounts of elemicin (21–39%) and some *trans*-iso-elemicin (0.2–0.4%), but in that case only low amounts of estragole (0.3%), as described by Lawrence [178]. Tarragon leaves are used as a domestic herb in soups and sauces such as “sauce bernaise”, pickled vegetables and salad dressings, baked goods, soft candy, meat products, and frozen dairy [176].

### 1.20. Ylang-Ylang

*Cananga odorata* Hook Fil. et Thomson. forma *macrophylla,* Annonaceae, is a large tree with fragrant flowers native to islands of tropical Asia. A similar species is *Canangium odoratum* Baill. forma *genuina* (=ylang-ylang) [1,179]. Ylang-ylang is one of the plants that are exploited at a large scale for its EO, which is an important raw material for the fragrance industry. The EOs extracted via steam distillation from the plant have been used mainly in the cosmetic industry but also in the food industry. A wide range of chemical compounds, including monoterpene, sesquiterpenes, and phenylpropanoids, have been isolated from this plant [180]. Generally, the EOs can be extracted from the aromatic plants by steam- or hydro-distillation. However, various combinations of extraction methods are necessary to extract all the volatile phytochemicals present in *Cananga odorata*. In addition to the steam- and hydro-distillation extraction methods, simultaneous steam-distillation-solvent extraction and supercritical fluid extraction (SFE) were also used to completely isolate most of the volatile secondary metabolites of the ylang-ylang flower. Following such extraction procedures, anethole (0.3–0.6%) as well as methyleugenol (0.2–1.5%) were detected in EOs of different Colombian origins [181,182]. Headspace Solid-phase Microextraction coupled with gas chromatography–mass spectrometry (HS-SPME-GC-MS) was used to characterize all the volatile compounds of *Cananga odorata* flower at different stages of development. The full-flowering stage appears to be the most suitable period for *Cananga odorata* flower harvest. Estragole was the only alkenylbenzene found. It occurered only in the developmental display-petal stage II (0.14%) and the full-flowering stage IV (0.13%; [183]). In contrast, Stashenko et al. found anethole (0.3%), iso-eugenol (1.0%), and methyleugenol (2.0%) in EOs, extracted from green flowers of intermediate maturity of Colombian ylang-ylang [184]. 161 individual compounds were identified in both low and high boiling fractions issued from distillation of mature ylang-ylang flowers. Amongst them, only traces of *trans*-anethole, eugenol, and iso-eugonal, but no safrole in EOs of ylang-ylang flowers were found [185]. None of the modern analytical techniques identified safrole as a constituent of ylang-ylang flower oils, in contrast to an earlier analysis using GC that assumed the presence of safrole and iso-safrole in commercial oils of *Cananga odorata* flowers [186]. In 1986, Gaydou et al. also reported safrole to be a component of the oxygenated fraction of the ylang-ylang EO, first grade, albeit to a very low amount of 0.1%, in addition to methyleugenol (0.2%) and eugenol (0.5%) [187].

## 2. Discussion

The most prominent examples of essential oils used in food and beverages are those produced from basil, fennel, tarragon, parsley, anis, star anis, nutmeg, and mace [188]. In general, plant-based powders, extracts, and oils used for flavoring of food are obtained from plant components by grinding and drying, hydro-distillation, steam-distillation, solvent extraction, supercritical fluid extraction, ultrasound- or microwave-assisted extractions, or a combination of diverse techniques [189,190,191]. The analytical data reviewed in this article demonstrate that the composition of EOs and extracts from plants is highly variable. This is not only due to the use of different species and variants but also dependent on geographic location, seasonal influences, and methods of extraction.

From a toxicological point of view, it is remarkable that plant-derived EOs and extracts often contain different potentially genotoxic and carcinogenic alkenylbenzenes, albeit they are frequently used for flavoring purposes in foods and beverages. Interestingly, parsley, commonly used as a culinary herb, was recently rendered the status “poisonous plant of the year 2023” by a botanical garden in Germany due to its content of myristicin and apiol [192]. Moreover, their usage in plant food supplements (PFS) may lead to exceptionally high exposure to alkenylbenzenes [125,145].

Due to the proven toxicological relevance, analytical studies of EOs are often focussing on the occurrence of the known genotoxic and carcinogenic alkenylbenzenes safrole, estragole, and methyleugenol [19,39,40]. However, as depicted in Table 1, it is expected that not-so-commonly known alkenylbenzenes, e.g., elemicin or apiols, also prevail in flavored products. Together, the aforementioned points speak in favor of the need for broader chemical analysis of alkenylbenzenes (and their combinations) in commercially used oils and extracts from herbs, spices, vegetables, and fruits, as well as in PFS, flavored foods, and beverages. Broader in the sense that a larger set of alkenylbenzenes should be taken into consideration.

Of note, the analytical method—especially the extraction procedure—may have an impact on the analytical results. Depending on the oil, extract, or food matrix, different sample preparation methods are currently in place to analyze the alkenylbenzenes [124,127]. For example, methods for analyzing ground nutmeg, wine and beer spices, and many food commodities are in place utilizing ultrasonic-assisted extractions in combination with solid phase extraction followed by GC-MS [193]. Another common method includes liquid-liquid microextraction of phenylpropenes from several different EOs, followed by GC-MS analysis [194]. Further approaches involve methanolic extracts from nutmeg powders that were analyzed by photodiodic array detection following ultra-high performance liquid chromatography (UHPLC) separation [125] or utilize technologies with functionalized magnetic microspheres for isolation of allyl-benzodioxoles such as myristicin and safrole from soft-drinks followed by GC-MS analysis [195].

Most recently, Dang and Quirino developed, described, and compared modern reversed-phase high-performance liquid chromatography (HPLC) and stacking-micellar electrokinetic chromatography (MEKC) methods for the determination of alkenylbenzenes in flavors [196,197]. They found that the analytical performance of HPLC was better than that of the stacking-MEKC method. Moreover, they concluded that hydro-distillation was applicable for the extraction of EOs, whereas extractions of complex food matrices deserve other techniques, such as liquid–liquid extraction, liquid-phase microextraction, or solid-phase extraction.

At least some alkenylbenzenes pose a potential genotoxic hazard. Consumer exposure to these compounds should therefore be as low as reasonably achievable. Noteworthy, it is so far not transparent to authorities in which food products, beverages, or PFS mixtures of alkenylbenzenes are present and to which quantities. This, in turn, hampers the assessment of potential health risks.

Standardized procedures for the quantifications of estragole, isosafrole, safrole, methyleugenol, and β-asarone in tea infusions via GC-MS are already established in Germany [198]. For estragole, another valid DIN-standard method exists in Germany for its quantification in fennel teas and other tea-like infusions [199]. Standardized analytical procedures for the determination of all other alkenylbenzenes, including myristicin, elemicin, parsley–apiol, dill–apiol, and their iso-forms, and others (Table 1), in many food matrices still need to be established and validated.

## 3. Conclusions

The data discussed in this article clearly show that the contents of toxic alkenylbenzenes, such as estragole, methyleugenol, and safrole, highly vary between different plants as well as in parts of the same plant used as food or for food manufacturing. Moreover, reliable analytical methods are needed for the assessment of further, less well-studied alkenylbenzenes, such as elemicin, apiols, and others, in relevant food matrices. In this context, it is important to quantify alkenylbenzenes not only in extracts and oils but also in final food products and beverages that are known to be flavored with oils and extracts from plants that are rich in alkenylbenzenes. Unless this endeavor is tackled sincerely, a realistic exposure assessment depending on consumer habits will be hampered.

## Figures and Tables

**Table 1:** Examples of structural formulas of alkenylbenzenes found in extracts and EOs mainly from various parts of herbs, spices, and some parts of vegetable plants (For a more detailed overview, see [1]).

*trans*-Anethole 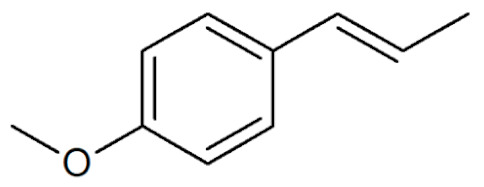	Anise, seeds (*Pimpinella anisum*); Cicely, leaf oils (Sweet chervil; *Myrrhis odorata*); Fennel (*Foeniculum vulgare);* Tarragon (*Artemisia dracunculus*)
Apiol from Dill 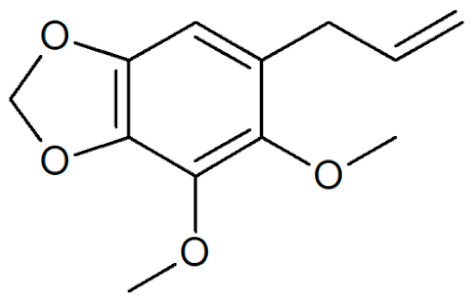	Dill (e.g., *Anethum graveolens* and *Anethum sowa*); Pepper, some variants (*Piper* spp.)
*trans*-Iso-Dill–Apiol 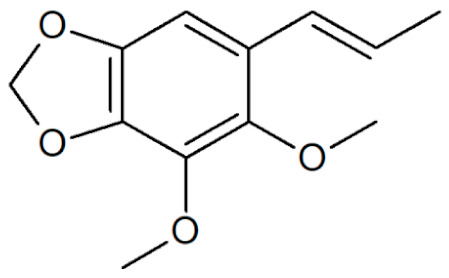	Dill (*Anethum graveolens* and *Anethum sowa*); Sea Fennel or Rock Samphire (*Crithmum maritimum*)
Apiol from Parsley 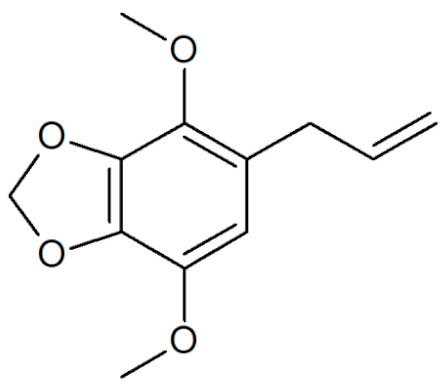	Parsley (*Petroselinum* spp.)
*trans-alpha-*Asarone (prohibited in the U.S.A.) 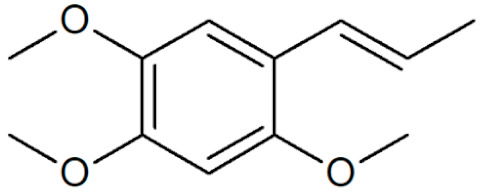	Flag, sweet (*Acorus calamus*)
*cis-beta*-Asarone (prohibited in the U.S.A.) (restricted §) 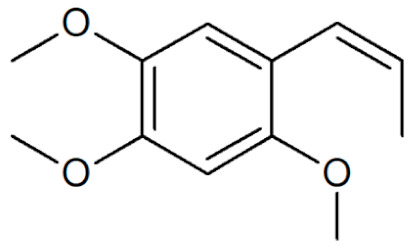	Flag, sweet (*Acorus calamus*)
*gamma*-Asarone (prohibited in the U.S.A.) 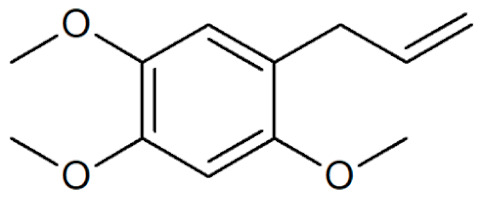	Flag, sweet, (*Acorus calamus*)
Carpacin 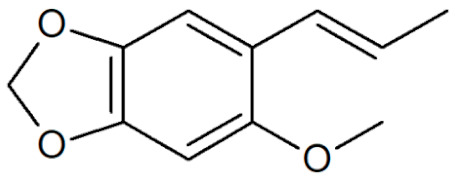	Carpano Tree, stem bark (*Cinnamomum* spp.); Waxflower, leaves (*Crowea exalata*)
Chavicol (4-Allylphenol) 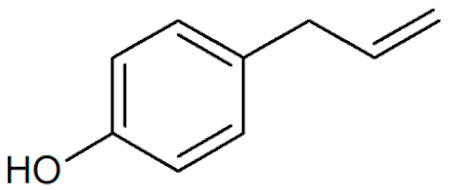	Betel, leaves (*Piper betle*); Bay, leaves (*Pimenta racemosa*)
Croweacin 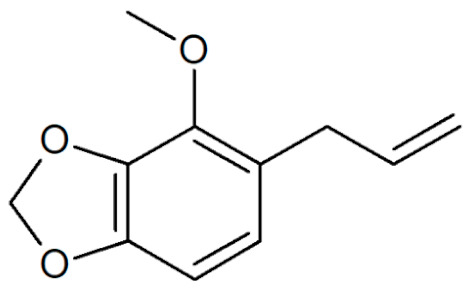	Waxflower, leaves (*Crowea exalata*)*;* Wild ginger, leaves (*Asarum hypogynum* and *A. costatum*)*;* Willow-leaved crowea, leaves (*Crowea saligna*)
Iso-Croweacin 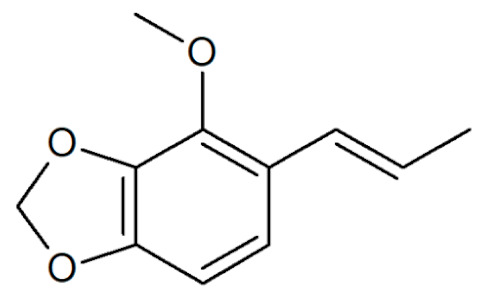	Waxflower, leaves (*Crowea exalata*)
Elemicin 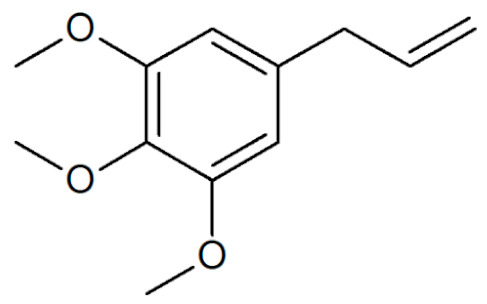	Carrot, wild, seeds (*Daucus carota* spp.)*;* Elemi, oils (*Canarium commune* and *C. luzonicum* and *C.* spp.); Nutmeg, kernel and mace (*Myristica fragrans*); Parsley seeds (*Petroselinum* spp.)
*trans*-Iso-Elemicin 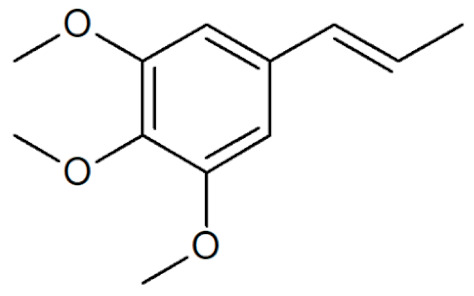	Nutmeg, kernel and mace (*Myristica fragrans*)
6-Methoxy-Elemicin 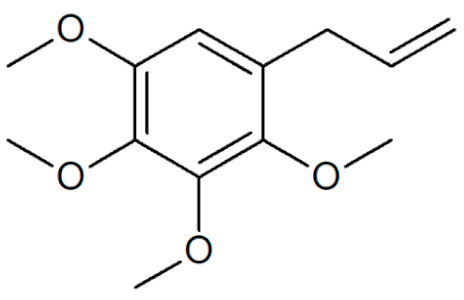	Parsley (*Petroselinum* spp.)
Estragole (Methylchavicol) (restricted §) 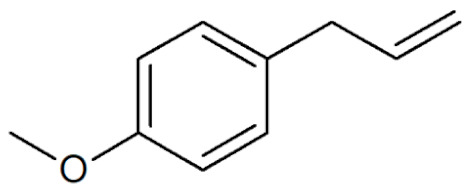	Anise, seeds (*Pimpinella anisum*); Basil, all parts (*Ocimum basilicum* spp.); Chervil (*Anthriscus cerefolium*); Fennel, seeds (*Foeniculum vulgare*); Tarragon (*Artemisia dracunculus*)
Eugenol 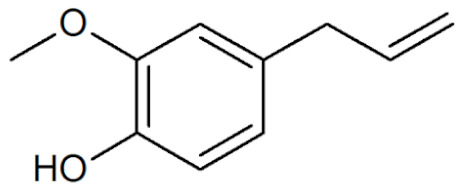	Basil, leaves (*Ocimum* spp.); Bay sweet, leaves (*Laurus nobilis*); Cloves (*Syzygium aromaticum*); Cinnamon, leaves (*Cinnamomum* spp.); Sweet bay, leaves (*Laurus nobilis*); Piment, berries and leaves (*Pimenta dioica, Pimenta racemosa*); Chervil, aerial parts (*Anthriscus cerefolium*); Cherries, sweet and sour, as well as many other fruits
*meta*-Eugenol (Chavibetol) 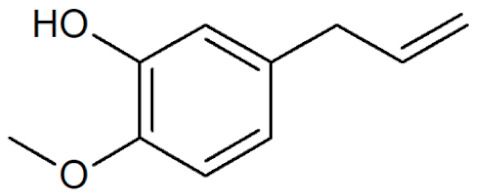	*Pimenta pseudocaryophyllus*, leaves (Brasil); Betel, leaves (*Piper betle*)
*ortho*-Eugenol 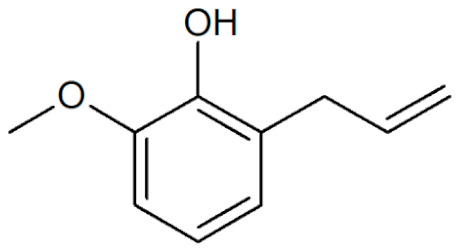	Dill (*Anethum graveolens* and *Anethum sowa*)
Acetyleugenol 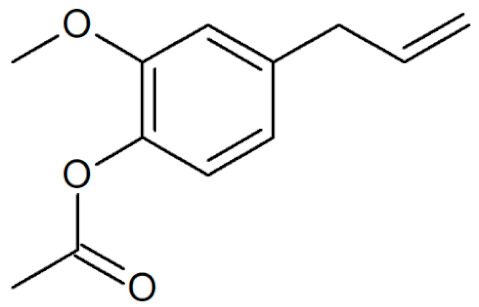	Basil, some variants (*Ocimum basilicum* spp.); Bay sweet, leaves (*Laurus nobilis*); Clove, flower buds and leaves (*Syzygium aromaticum*)
*trans*-Iso-Eugenol 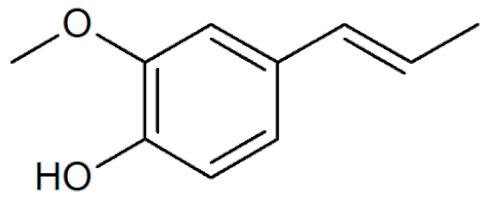	Ylang-ylang, tree flowers (*Canangium odoratum genuine*)
*trans-pseudo-*Iso-Eugenyl-2-methylbutyrate 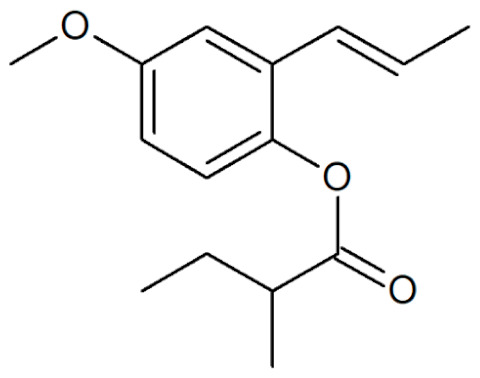	Anise, seeds (*Pimpinella anisum*)
6-Methoxyeugenol 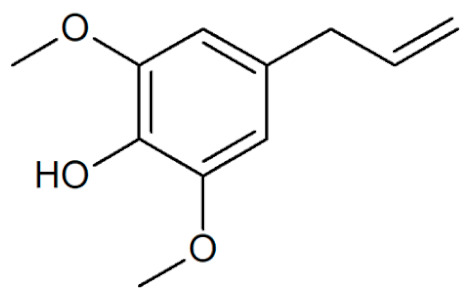	Nutmeg, kernel and mace (*Myristica fragrans*)
Exalatacin 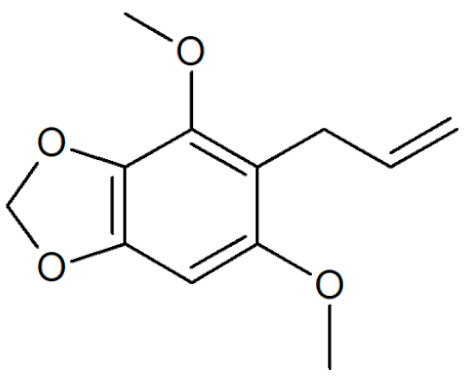	Waxflower, leaves (*Crowea exalata)*
Methyleugenol (restricted §) 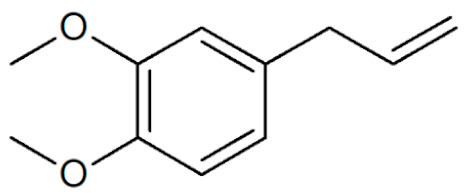	Basil, all parts (*Ocimum* spp.); Bay sweet, leaves (*Laurus nobilis*); Carrot seed, oils (*Daucus carota* spp.); Cinnamon, all parts (*Cinnamomum* spp.); Citronella, oils (*Cymbopogon winterianus*); Cloves, flower buds (*Syzygium aromaticum*); Nutmeg, kernel and mace (*Myristica fragrans*); Ysop (*Hyssopus officinalis*)
*trans*-Iso-Methyleugenol 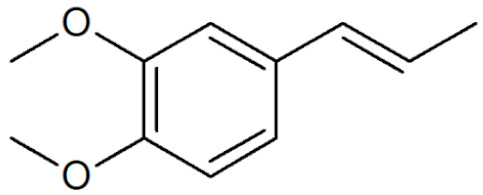	Carrot, seeds of some variants (*Daucus carota* spp.); Citronella, oils (*Cymbopogon winterianus*)
Myristicin 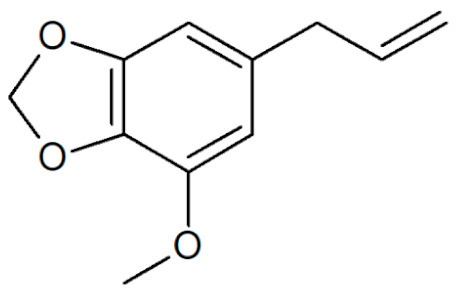	Anise, seeds (*Pimpinella anisum*); Carrot, seeds and roots of some variants (*Daucus carota*); Dill, oils (*Anethum graveolens* and *Anethum sowa*); Fennel (*Foeniculum vulgare);* Nutmeg, kernel and mace (*Myristica fragrans*); Parsley, leaves and seeds (*Petroselinum* spp.); Parsnip, roots (*Pastinaca sativa*)
*trans*-Iso-Myristicin 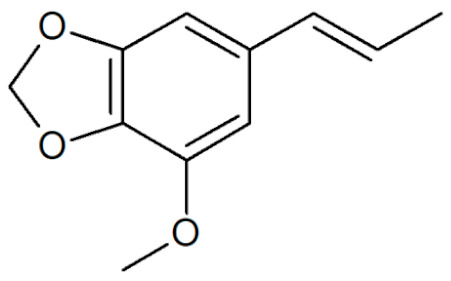	Nutmeg, kernel and mace (*Myristica fragrans*); Parsley (*Petroselinum sativum*); Dill (*Anethum graveolens* and *Anethum sowa*)
*trans*-Nothosmyrnol 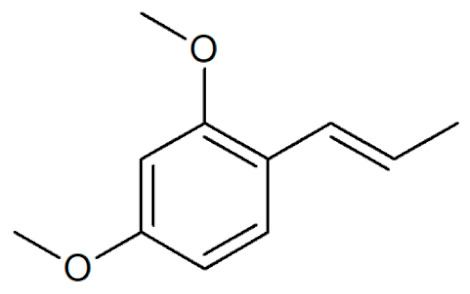	Chervil (*Anthriscus cerefolium*)
Osmorhizole 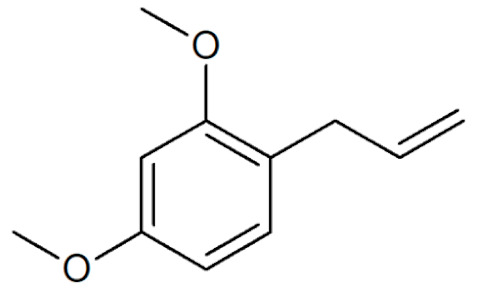	Chervil (*Anthriscus cerefolium*)
Safrole (prohibited in the U.S.A.) (restricted §) 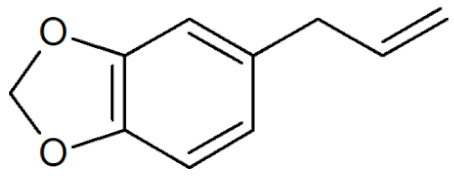	Anise, seed (*Pimpinella anisum*); Camphor tree; wood branches and leaves (*Camphora officinarum* and *Cinnamomum camphora*); Cinmamon, leaves (*Cinnamomum zeylanicum* and *C. cassia*) Fennelwood tree, roots (*Sassafras albidum*); Nutmeg, leaves, kernel and mace (*Myristica fragrans*); Sweetwood, stem bark (*Ocotea opifera*); *Piper auritum*, leaves; *Piper divaricatum*, all parts; *Piper hispidinervum*, leaves
*trans*-Iso-Safrole (prohibited in the U.S.A.) 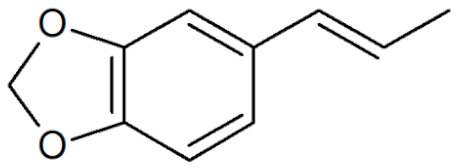	Nutmeg, kernel and mace (*Myristica fragrans*); Pepper, several parts and variants (*Piper* spp.); Ylang-ylang, tree flower (*Canangium odoratum genuine*)
Sarisan 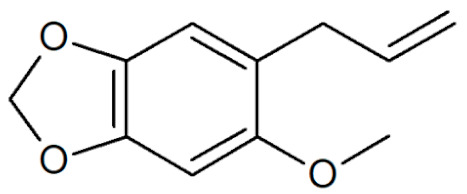	Alpine Lovage, roots (*Ligusticum mutellina*); Ginger, wild, roots (*Asarum heterotropoides*); Sweetwood, stem bark (*Ocotea opifera*); Pepper, several (*Piper* spp., e.g., EOs from ripe berries of *Piper guineense,* from roots of *Piper auritum,* from leaves of *Piper solmsianum*, *Piper callosum*, *Piper affinis hispidinervum*, and of *Beilschmiedia miersii*)

§ restricted according to Regulation (EC) No 1334/2008 (consolidated version 2022).

**Table 2:** Herbs, spices, and vegetables that have been identified to contain alkenylbenzenes. Mentioned are those alkenylbenzenes (Ab) assumed to predominate in essential oils.

Common Name	Species	Family	Ab 1 in EO	Ab 2 in EO
Allspice	*Pimenta dioica*	Myrtaceae	Methyleugenol 0.1–82% in EO from berries	Eugenol 8–95% in EO from berries
Anise	*Pimpinella anisum*	Apiaceae	*trans*-Anethole 80–90% in EO from dried ripe fruits	Estragole 1–8% in EO from dried ripe fruits
Chinese Star Anise	*Illicium verum*	Illiciaceae	*trans*-Anethole 88–92% in EO from dried ripe fruits	Estragole 0.5–5.5% in EO from dried ripe fruits
Japanese Star Anise	*Illicium anisatum*	Illiciaceae	Methyleugenol 9.8% in EO from dried ripe fruits	Safrole 6.6% in EO from dried ripe fruits
Basil, sweet	*Ocimum basilicum*	Lamiaceae	Methyleugenol 9–87% in EO of all aerial parts	Estragole 0–58% in EO of all aerial parts
Bay	*Pimenta racemosa*	Myrtaceae	Eugenol 0–66% in EO from leaves	Methyleugenol 0–64% in EO from leaves
Bay, sweet	*Laurus nobilis*	Lauraceae	Methyleugenol up to 21% in EO from wild leaves	Elemicin up to 5% in EO from wild leaves
Carrot	*Daucus carota sativa*	Apiaceae	Myristicin in EO from carrot roots (40 ppm)	Myristicin in EO from carrot roots (0.5–15 ppm)
Carrot, wild	*Daucus carota sahariensis*	Apiaceae	Myristicin 44% in EO from carrot roots	Myristicin 34% in EO from carrot leaves
Chervil	*Anthriscus cerefolium*	Apiaceae	Estragole 75–80% in EO from aerial parts	1-Allyl-2,4-dimethoxybenzene unknown quantity
Cinnamon	*Cinnamomum ceylanicum*, and *C. cassia*	Lauraceae	Eugenol up to 87% in EO from leaves	Safrole (traces) in EO from bark
Clove	*Syzygium aromaticum*	Myrtaceae	Eugenol up to 89% in EO from air-dried buds	Acetyleugenol up to 7% in EO from air-dried buds
Dill	*Anethum graveolens*	Apiaceae	Dill–Apiol up to 25% in EO from dill seeds and also from roots (up to 67%)	Myristicin 0.2% in EO from seeds and 4.4% in EO from roots
Dill	*Anethum sowa*	Apiaceae	Dill–Apiol 12–15% in EO from dill seeds	Myristicin 0.2% in EO from dill seeds
Elemi	*Canarium commune*	Burseraceae	Elemicin in oleoresins from distillations of resinous exudations from trees	Not yet defined
Fennel	*Foeniculum vulgare* var. *dulce*	Apiaceae	*trans*-Anethole 60–90% or up to 88% estragole in EO from seeds and other aerial parts	Dill–apiol up to 94% in EO from fennel roots
Lemon Balm	*Melissa officinalis*	Lamiaceae	Estragole 6,3% in EO from leaves	Eugenol 3.5–4.5% Eugenylglucoside in EO from dried leaves or flowers
Cylon Lemon Grass	*Cymbopogon nardus*	Poaceae	Iso-Methyleugenol 7.2% in EO from leaves	Methyleugenol 1.7% in EO from leaves
Lovage	*Levisticum officinale*	Apiaceae	*trans*-Anethole 0.3% in EO from aerial parts	Not identified
Nutmeg	*Myristica fragrans*	Myristicaceae	Myristicin 0.5–12.4% in EO from kernels	Elemicin 0.3–4.6% in EO from kernels
Parsley	*Petroselinum sativum,* and *P. Crispum*	Umbelliferae	Myristicin 20.3–94.1% in EO from seeds	Parsley–apiol 0.6–78.7% in EO from seeds
Pepper	*Piper nigrum*	Piperaceae	Eugenol 10–120 mg/kg in EO from berries	Estragole 2.2–46 mg/kg in EO from berries
Pepper	*Piper guineense*	Piperaceae	Myristicin 17% in EO from berries	Sarisan 16% in EO from berries
Purple Haze	*Agastache foeniculum*	Lamiaceae	Estragol 40–93% in EO from aerial parts	Methyleugenol 0.2–44% in EO from aerial parts
Tarragon	*Artemisia dracunculus*	Asteraceae	Estragole up to 69% in EO from leaves	Methyleugenol up to 28% in EO from seeds
Ylang-ylang	*Canangium odoratum genuine*	Annonaceae	*trans*-Anethole 0.3–0.6% in EO from flowers	Methyleugenol 0.2–1.5% in EO from flowers

(EO) essential oil; (Ab1) Highest amounts of an alkenylbenzene reported in EOs; (Ab2) Second highest amounts of an alkenylbenzene reported in EOs; % values are described and referenced in the main text.
